# Effects of genotypes on sperm quality and fertility of buffalo bulls

**DOI:** 10.5455/javar.2025.l961

**Published:** 2025-09-22

**Authors:** Md. Faizul Hossain Miraz, Gautam Kumar Deb, Sheikh Mohammad Jahangir Hossain, Shahrina Akter

**Affiliations:** 1 Biotechnology Division, Bangladesh Livestock Research Institute (BLRI), Savar, Dhaka, Bangladesh; 2 Graduate School of Integrated Sciences for Life, Hiroshima University, Hiroshima, Japan

**Keywords:** Buffalo, genotypes, cryopreservation, kinematics, motility, semen

## Abstract

**Objectives:**

The objective of this study was to evaluate the effects of genotypes on semen quality and fertility of Murrah, Nili-Ravi, and Bangladeshi indigenous buffalo bulls.

**Materials and Methods:**

Fresh semen was collected from pure Murrah, Nili-Ravi, and indigenous buffalo bulls. Sperm concentration, motility, morphology, kinematics, and dose/ejaculation were assessed by a computer-assisted sperm analyzer. Fresh semen was cryopreserved, and post-thaw semen quality and fertility were evaluated. The hypoosmotic swelling test (HOST) was used to evaluate plasma membrane integrity.

**Results:**

Indigenous bulls produce a lower volume of semen but exhibit higher total motility than Murrah and Nili-Ravi bulls. After cryopreservation, the post-thaw motility remains consistent, except for significant motility losses observed in Nili-Ravi bulls. Nili-Ravi bulls also showed a higher incidence of bent and coiled tails, while indigenous bulls exhibited significantly lower percentages of distal droplets than the others. Static sperm have significantly smaller head width, head elongation, and head area, and higher tail straightness than motile and progressively motile sperm. Fresh indigenous bull sperm have higher average path velocity, straight linear velocity, curvilinear velocity, and linearity than others. The Nili-Ravi bull has significantly lower HOST-positive sperm than others in fresh and post-thaw conditions. Genotypes did not exhibit any significant differences in dose/ejaculation and fertility rate.

**Conclusion:**

Indigenous bulls exhibit superior semen quality in motility, plasma membrane integrity, and post-thaw viability compared to Murrah and Nili-Ravi bulls. Additionally, the higher progressive motility and improved kinematic properties observed in indigenous bulls may have contributed to their higher fertility rates. However, no significant differences in fertility outcomes were found among the genotypes, suggesting that all three genotypes performed similarly in terms of fertility through artificial insemination (AI), at least under on-station conditions. Further research needs to be carried out to evaluate AI efficiency and fertility rate under on-farm conditions.

## Introduction

The livestock industry of Bangladesh has consistently experienced steady growth over the past few decades, driven by farmer-friendly government policies and collaborative efforts from both farmers and entrepreneurs. The availability of milk per capita has improved recently; now, 193.38 ml of milk per person per day is available, compared to the FAO’s recommended quantity of 250 ml [1]. India continues to be the largest producer of milk in South Asia, accounting for almost 75% of the total supply, followed by Pakistan [2]. Bangladesh’s growing dairy sector contributes a smaller but significant portion to the regional total [2]. About 90% of the total milk is produced by about 24.5 million cattle, 3%–4% from 1.5 million buffalo heads, and the remaining 4% comes from goats [3]. Similarly, buffalo contributes only 0.1 million metric tons to the country’s total meat production, compared to 0.4 million metric tons from cattle [4]. These statistics explore leveraging buffalo potential for enhanced milk and meat production, thereby increasing overall productivity. However, the major challenge lies in the lower milk production (600–1,000/l, 250–270-day lactation period) potentialities of native buffalo [4]. Studies are being conducted to boost the productivity of native buffalo. Nevertheless, Bangladesh currently lacks a specialist buffalo breed with a higher potential for milk and meat production. Therefore, there are two ways to increase the production of buffalo: either by selective breeding within the native population or by crossbreeding with improved buffalo breeds.

Milk and meat production of indigenous buffaloes through genetic improvement by crossbreeding has gained much attention in Bangladesh. Murrah and Nili-Ravi are the two most promising breeds of India that are known for their higher milk yield and quality. Superior breeding males of the Murrah and Nili-Ravi breeds of buffalo have been imported from India to produce frozen semen for crossbreeding with native buffalo. To have a successful crossbreeding program, breeding bulls with proven pedigrees must produce high-quality semen, since bull fertility and semen-related characteristics are essential to preserve overall herd fertility [5]. Bull fertility markers include sperm quality characteristics such as motility, morphology, concentration, and freezability [6]. However, the availability of high-quality breeding bulls and semen remains a significant constraint. There are several functional and intrinsic limitations of frozen semen production. Additionally, the natural limitations that make the matter worse than cattle include smaller testicles, less quantity of semen produced, and an epididymal sperm reserve in buffalo bulls [6,7].

Given these challenges, genotypes and breed differences are other important predictors of sperm quality and fertility in different animal species. Studies have demonstrated that there might be significant differences among buffalo bull genotypes in different sperm parameters, including motility, morphology, viability, and seminal plasma composition [8]. For instance, Murrah and Nili-Ravi buffalo bulls differed significantly in sperm motility characteristics [8]. While male and female factors both contribute to successful fertilization, semen analysis remains the most frequently used diagnostic tool to assess male reproductive potential due to its relatively high accuracy and cost-effectiveness [9]. Therefore, this study compares the differences in sperm quality and fertility potentialities of Murrah, Nili-Ravi, and indigenous buffalo genotypes for their suitability in crossbreeding programs in Bangladesh.

## Materials and Methods

### Ethical approval

Bangladesh Livestock Research Institute’s (BLRI) “Animal Experiment Ethics Committee” granted necessary ethical approval for conducting the experiment (approval number 586).

### Buffalo bull selection and management

Buffalo breeding bulls, aged 3 to 5 years, were housed individually in an intensive housing system with uniform management throughout. Pure Murrah and Nili-Ravi bulls were imported from India, and an indigenous buffalo bull was used from the buffalo breeding stock of the BLRI. Imported bulls were allowed a 6-month adjustment period before using their semen for the experiment. The study includes 45 ejaculates from nine breeding bulls (3 bulls/group) of three genotypes of buffalo (Murrah, Nili Ravi, and Indigenous buffalo).

### Semen collection and evaluation

**Figure 1.**
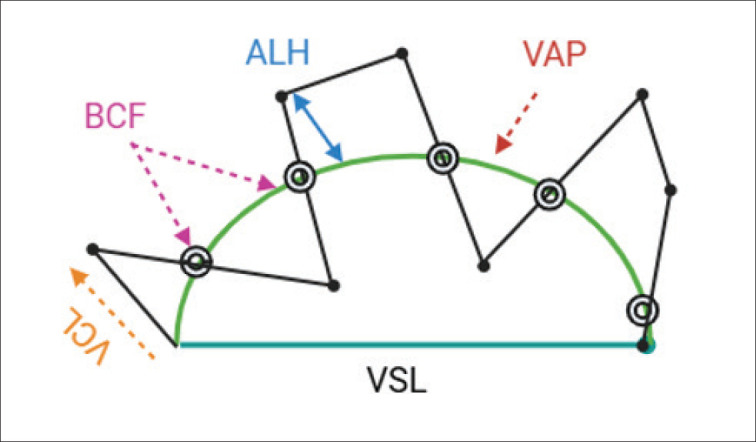
Sperm kinematics measured by CASA.

Semen was routinely collected once a week in the early hours of the morning (6–6.30 AM) using an artificial vagina set. Collected semen was transferred to the laboratory for analysis and processing. A computer-assisted sperm analyzer (CASA) (Hamilton Thorne, IVOS II) was used to quantify motility, morphology, concentration, and kinematics [10]. In brief, a semen (3 μl) sample was put onto a preheated Leja^®^ slide for sperm motility and kinematics analysis. Average path velocity (VAP), straight linear velocity (VSL), curvilinear velocity (VCL), amplitude of lateral head displacement (ALH), straightness (STR), linearity (LIN), and beat cross frequency (BCF) were measured for kinematic properties (Fig. 1). CASA was set as follows: frame rate-60 Hz, frames acquired-30, minimum contrast-35, cell size-9 (pixels), cell intensity-110 (pixels), VAP-50 μm/sec, STR-70%, VAP cut-off-30 μ/sec, and VSL cut-off-15 μ/sec.

### Semen dilution, equilibration, and freezing

AndroMed (Cat no. 13503/0200, Minitube, Germany) diluter was used at a 1:4 (Andromed: Mili-Q water) ratio to dilute fresh semen from three genotypes of bulls to get a final concentration of 80 × 10^6^ spermatozoa/ml. Equilibration was conducted for 4 h at 4°C in a cold handling cabinet (Minitube, Germany). A French mini-straw (0.25 ml) (20 million spermatozoa/straw) was filled with diluted semen and sealed using an automated filling and sealing machine (MPP Uno, Minitube, Germany). Liquid nitrogen vapor was used to freeze the diluted semen using a programmed bio-freezer (Turbo Freezer M, Minitube, Germany). The frozen semen was then placed in liquid nitrogen (−196°C) for storage.

### Post-thaw evaluation and artificial insemination (AI)

Frozen semen straw was evaluated post-thaw (37°C, 15 sec) following a 24-h storage period. Motility, morphology, and kinematic characteristics of frozen semen samples from Murrah, Nili-Ravi, and indigenous bulls were evaluated by CASA. Indigenous buffalo cows at their second or third parties were inseminated artificially at their natural estrous condition. After 60 days of insemination, pregnancy was confirmed by rectal palpation, and the non-return rate was calculated. 


$$\mathrm{Non}-\mathrm{return}\;\mathrm{rate}\;\mathrm{(\%)}\;=\;\frac{\mathrm{Number}\;\mathrm{of}\;\mathrm{buffaloes}\;\mathrm{pregnant}\;\mathrm{after}\;\mathrm{inse}\min\mathrm{ation}}{\mathrm{Tota}l\;n\mathrm{umber}\;\mathrm{of}\;\mathrm{services}}\;\times \;100$$

### Hypoosmotic swelling test (HOST)

Sperm plasma membrane integrity was evaluated by the HOST. Sperm samples were incubated in sodium citrate and fructose solution (150 mOsm/l) for 30 min at 37°C. After incubation, the reaction was stopped by adding 10% formalized eosin (5 µl). The stained sample was fixed and observed by a phase contrast microscope. About 200 sperm were observed for each genotype from different microscopic fields.

### Statistical analysis

The data were tested for normality using the Shapiro–Wilk test and for homogeneity of variances using Levene’s test. Semen volume, sperm concentration, morphology, kinematic parameters, plasma membrane integrity, fertility, and dose per ejaculate among the three genotypes were compared. Differences between fresh and frozen semen were also evaluated. Statistical analysis was performed using one-way ANOVA followed by Tukey’s *post-hoc* test for multiple comparisons. A *p*-value less than 0.05 was considered statistically significant. All analyses were conducted using GraphPad Prism software (version 8.0.2).

## Results

### Semen volume and sperm concentration

**Figure 2.**
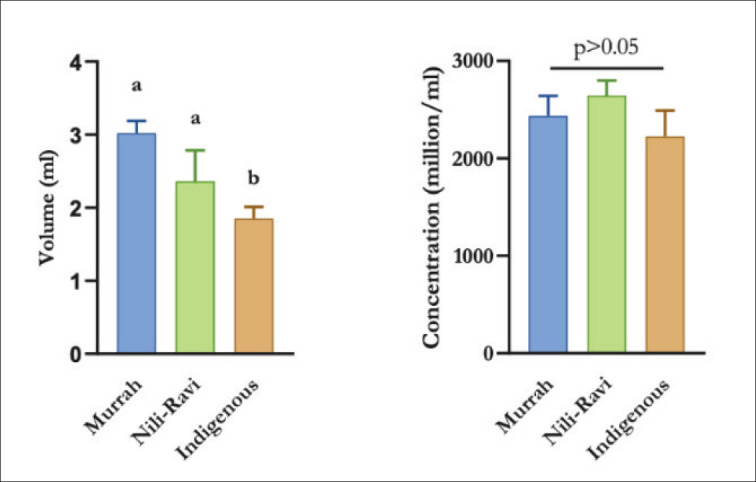
Volume (ml) and concentration (million/ml) of Murrah, Nili-Ravi, and Indigenous bull. Values are expressed as mean and standard deviation (SD).

Freshly ejaculated semen samples of three genotypes of the bull were evaluated by CASA. Indigenous bulls produced significantly less volume of semen compared to Murrah and Nili-Ravi bulls; however, no significant difference was observed between Murrah and Nili-Ravi bulls (Fig. 2). Sperm concentration, however, did not show any significant differences among the genotypes (Fig. 2).

### Sperm morphology assessment

**Figure 3.**
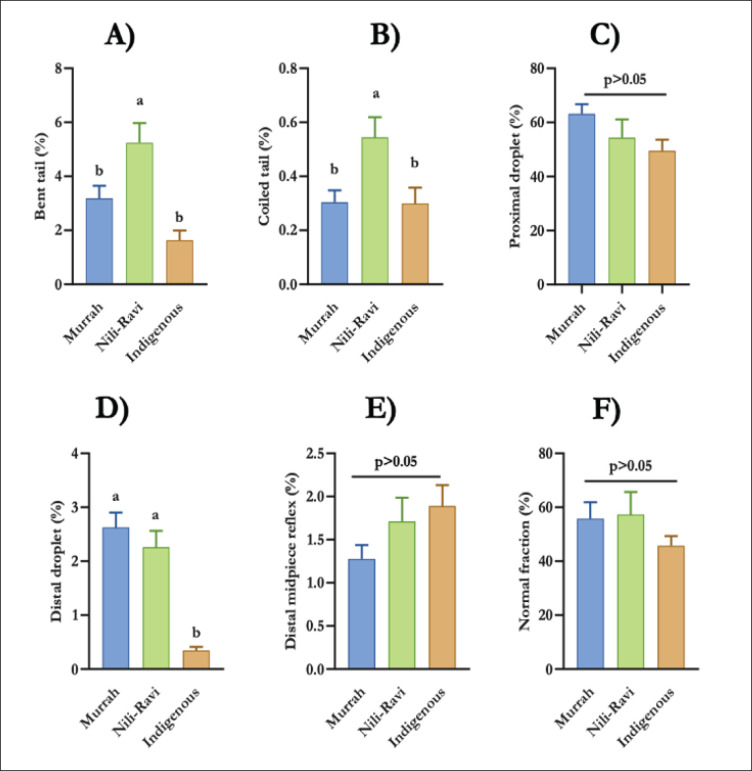
Sperm morphology (mean ± SD) of Murrah, Nili-Ravi, and Indigenous bulls (A-Bent tail%, B-Coiled tail (%), C-Proximal droplet (%), D-Distal droplet (%), E-Distal mid-piece reflex (%), F-Normal fraction).

In addition to evaluating semen volume and sperm concentration, sperm morphology was assessed to further characterize the quality and fertilization potential of semen across the three bull genotypes. Sperm from Nili-Ravi bulls exhibited a higher percentage of bent tails and coiled tails compared to Murrah and indigenous bulls (Fig. 3A and B). In contrast, indigenous bulls had significantly lower distal droplet percentages than both Murrah and Nili-Ravi bulls (*p *< 0.05) (Fig. 3D). No significant differences were found among the genotypes for proximal droplets (%), distal midpiece reflexes (%) and the normal sperm fraction (%) (*p* > 0.05) (Fig. 3C, E, F).

### Sperm motility and effects of cryopreservation

**Figure 4.**
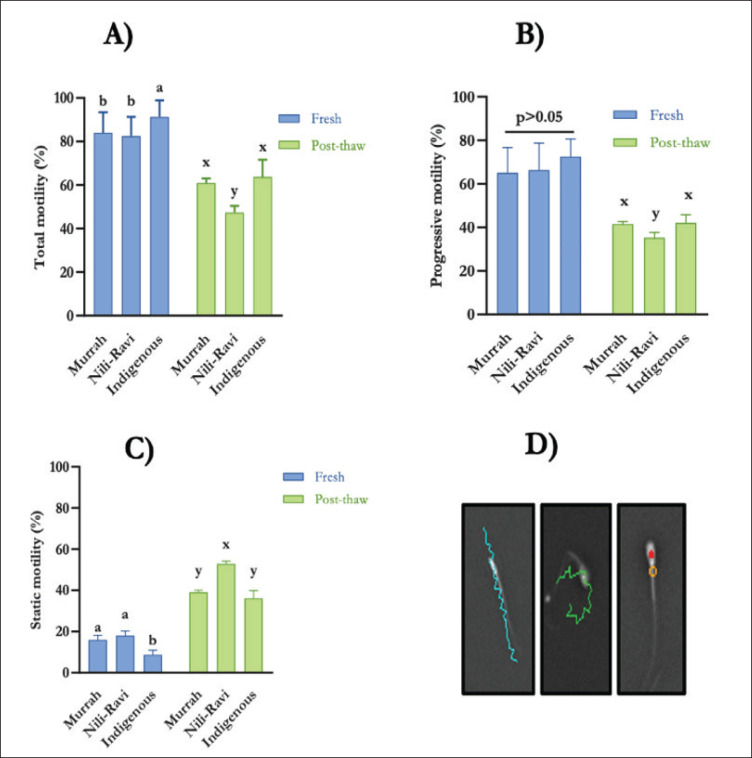
Fresh and post-thaw motility (mean ± SD) of Murrah, Nili-Ravi, and Indigenous bulls (A-Total motility (%), B-Progressive motility (%), C-Static motility (%), D- Motility (Aqua color: Progressive sperm, Green color: Motile sperm, Red color: Static sperm)).

Sperm motility is one of the most important parameters to gain a comprehensive understanding of semen quality and its resilience to cryopreservation. In fresh semen, there were significant differences in total and static motility among the genotypes; however, the progressive motility was quite similar. Indigenous bulls have significantly higher total motility and lower static motility than Murrah and Nili-Ravi bulls (*p* < 0.05) (Fig. 4A and C). Cryopreservation reduces total and progressive motility and increases static motility irrespective of genotypes. In post-thaw semen, Nili-Ravi bulls have lower total and progressive motility and higher static motility than others (*p* < 0.05), which indicates that Nili-Ravi bulls are more affected by cryopreservation in this experimental condition (Fig. 4A–C).

### Sperm morphometry

Sperm morphometry of Murrah, Nili-Ravi, and indigenous buffalo sperm was evaluated to understand whether the morphometry has any relationship with sperm motility. Static sperm have a significantly smaller head width, head elongation, and head area than motile and progressively motile sperm. Although tail length did not differ among different motility groups, static sperm had higher tail STR than motile and progressive sperm (Table 1). There were no significant differences in sperm head length, head width, head elongation, head perimeter, head area, tail length, and tail STR among the genotypes (Table 2).

### Sperm kinematic parameters

**Figure 5.**
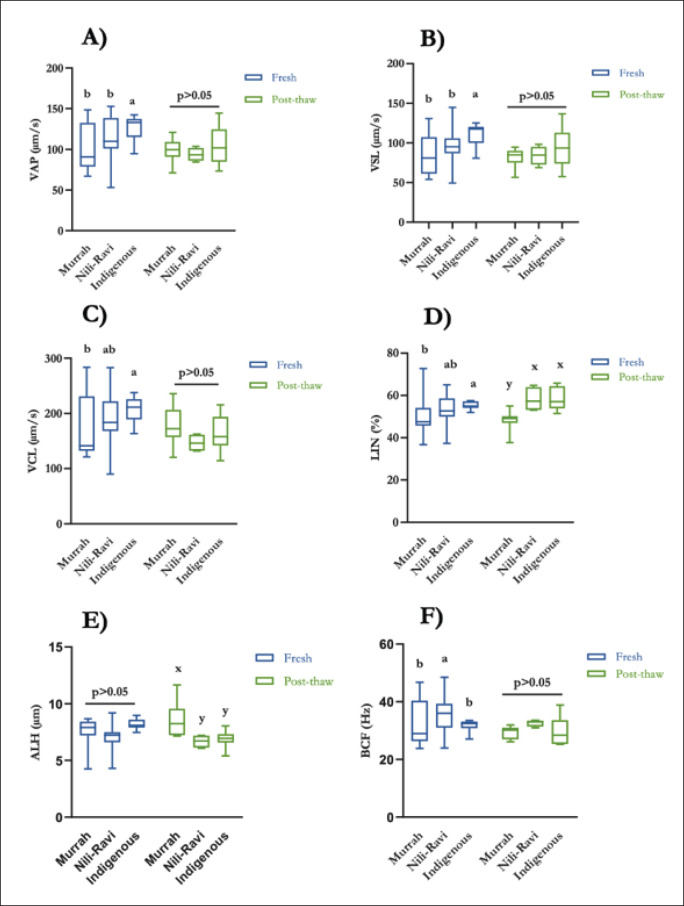
Sperm kinematics of fresh and post-thaw sperm of Murrah, Nili-Ravi, and Indigenous bulls based on total motile sperm (A-VAP (µm/sec), B-VSL (µm/sec), C-VCL (µm/sec), D-LIN (%), E-ALH (µm), and F-BCF (Hz)).

Sperm kinematics measure the distance of each head point of an individual sperm at the time of acquisition, and these parameters can focus on individual sperm of a subpopulation. In fresh semen, significantly higher VAP, VSL, VCL, and LIN were observed in Indigenous bulls (*p* < 0.05) (Fig. 5A–D). Following cryopreservation, significantly higher ALH and lower LIN were observed in Murrah bulls (*p* < 0.05) (Figs. 5D and E), and other kinematic parameters remain insignificant among the genotypes.

### Plasma membrane integrity

**Figure 6.**
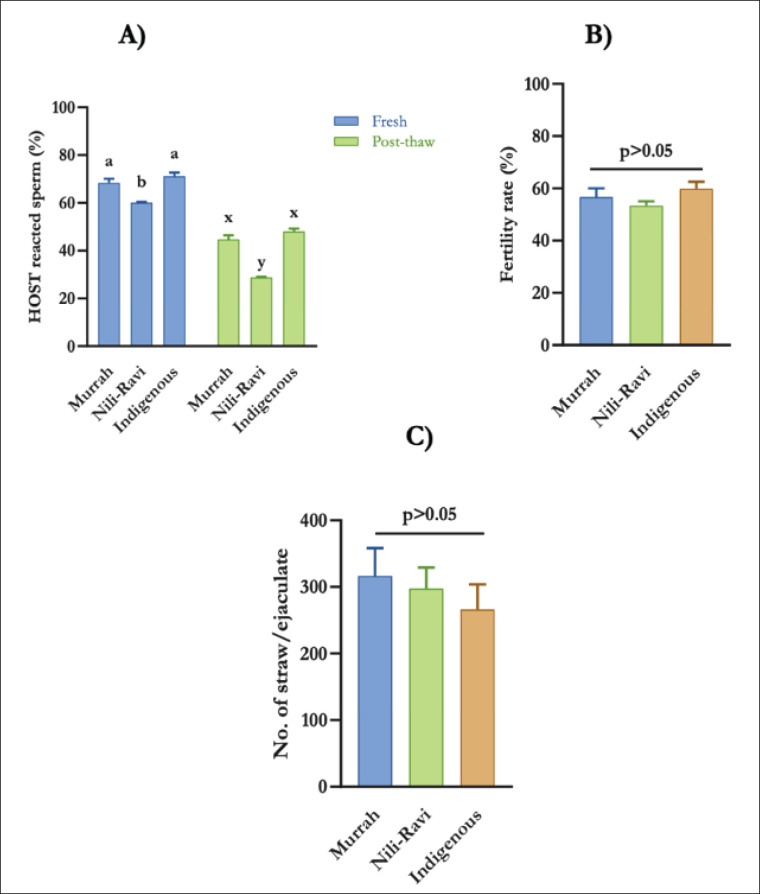
Plasma membrane integrity (A), fertility (B), and No. of straw/ejaculate (C) of Murrah, Nili-Ravi, and Indigenous buffalo bulls. Values are expressed as mean and standard deviation (SD).

The sperm plasma membrane serves as a protective barrier against physiological stress and extracellular damage, and its integrity is closely associated with sperm motility and fertilization potential. The HOST was used to assess plasma membrane integrity, with a higher percentage of HOST-positive sperm indicating better membrane integrity. Nili-Ravi bulls showed a significantly lower percentage of HOST-positive sperm compared to Murrah and Indigenous bulls in both fresh and post-thaw semen (*p* < 0.05) (Fig. 6A).

### Comparative semen production performances

The total number of frozen semen doses produced per ejaculate was compared among the three bull genotypes. The genotypes did not exhibit any differences; nonetheless, Murrah bulls were the most productive (300.6 ± 94.71) in terms of doses/ejaculation produced, followed by Nili-Ravi (279.9 ± 85.86) and Indigenous bulls (255.1 ± 90.53) (Fig. 6C). Given that 52.14 ejaculates (once per week, 52.14 weeks/year) are projected to be frozen per bull per year, the average frozen semen doses per year per bull for Murrah, Nili-Ravi, and Indigenous bulls might be 15,675.60 ± 49, 14,595.75 ± 4476.58, and 13,301.88 ± 47, respectively.

### Fertility outcomes following AI

Frozen-thawed semen from three genotypes of bulls was used for AI in 78 indigenous buffalo cows (Murrah-27, Nili-Ravi-24, and Indigenous-27) at the BLRI buffalo research farm. Following pregnancy evaluation at 60 days post-AI, there were no significant differences observed in bull fertility among the genotypes (*p* > 0.05) (Fig. 6B).

**Table 1.** Head and tail morphometric characteristics of motile, progressive, and static sperm.

Parameter	Motile	Progressive	Static
Head length (µm)	9.72 ± 0.59	9.47 ± 1.40	9.71 ± 2.14
Head width (µm)	4.69^a^ ± 0.28	4.50^a^ ± 0.37	4.09^b^ ± 0.0.59
Head elongation	0.50^a^ ± 0.04	0.50^a^ ± 0.08	0.45^b^ ± 0.10
Head perimeter(µm)	25.26 ± 1.19	24.63 ± 2.60	24.58 ± 4.53
Head area(µm^2^)	30.37^a^ ± 2.75	27.91^ab^ ± 5.13	25.48^b^ ± 7.09
Tail length(µm)	25.34 ± 2.49	24.72 ± 4.35	25.68 ± 6.20
Tail STR	80.09^b^ ± 3.93	79.49^b^ ± 3.90	85.13^a^ ± 7.35

Mean values in the same row with different superscripts (^a, b, c^) differ significantly (*p* < 0.05). Values are expressed as mean and standard deviation (SD).

**Table 2.** Head and tail morphometric characteristics of Murrah, Nili-Ravi, and indigenous buffalo sperm.

Parameter	Murrah	Nili-Ravi	Indigenous
Head length (µm)	9.56 ± 1.05	9.57 ± 1.13	9.59 ± 1.65
Head width (µm)	4.60 ± 0.34	4.34 ± 0.35	4.56 ± 0.54
Head elongation	0.50 ± 0.0.62	0.48 ± 0.66	0.50 ± 0.08
Head perimeter(µm)	24.86 ± 2.05	24.80 ± 2.13	24.74 ± 3.42
Head area(µm^2^)	29.13 ± 4.26	28.82 ± 4.34	27.04 ± 6.95
Tail length(µm)	25.93 ± 3.54	24.82 ± 3.90	25.38 ± 4.20
Tail STR	79.35 ± 4.16	80.12 ± 4.13	82.44 ± 7.67

Values are expressed as mean and standard deviation (SD).

## Discussion

The purpose of the spermatozoa is to produce viable zygotes and live offspring through successful fertilization [11]. After ejaculation, the spermatozoa pass through the complex physical barrier of the female reproductive tract to reach the fertilization site [12]. Sperm morphology, motility, and functional integrity are the most important attributes of sperm for successfully passing the events of acrosome reaction, capacitation, hyperactivation, and fertilization [13]. Sperm fertilization capacity may be affected by the differences in sperm quality across various breeds or even between bulls within the same breed. Our research offers valuable insights into the impact of genotype on semen quality traits in buffalo bulls, examining how these traits may influence their fertility outcomes.

Our findings demonstrated substantial genotypic influences on semen volume and morphology affecting overall semen quality. Indigenous bulls produce reduced semen volumes compared to Murrah and Nili-Ravi bulls. This disparity is presumably attributable to the increased body size and generally bigger testicular dimensions of the Murrah and Nili-Ravi breeds, which might facilitate enhanced spermatogenesis [14]. Murrah bull semen volumes in our study were lower than some reported values (e.g., 4.48 ml) [15]. Individual variations and the lower number of bulls studied could explain these variations. Notably, the indigenous buffalo bull produces semen that is identical in volume and concentration to that observed in our previous study [16]. Beyond volume, sperm morphology exhibited distinct patterns, irrespective of genotype. Nili-Ravi bulls demonstrated a greater prevalence of bent and coiled tails, but indigenous bulls typically exhibited fewer abnormalities, notably a reduced occurrence of distal droplets. This suggests that indigenous buffalo, having adapted to the local environment, may exhibit fewer morphological abnormalities due to evolutionary adaptations. In contrast, imported Murrah and Nili-Ravi bulls may experience physiological stress when adapting to new environmental conditions, perhaps resulting in higher abnormalities. Climatic stress is recognized to cause cellular damage through elevated formation of reactive oxygen species (ROS), impacting tail defects and droplet percentages [14]. Despite all three genotypes surpassing the overall total abnormality standards (often below 15%), their tail deformities remained within the permissible range (below 10%) for frozen semen production [17].

The influence of genotype on sperm motility and resistance to cryopreservation was notably apparent. Indigenous bulls show superior total and progressive motility and reduced static motility in fresh semen compared to Murrah and Nili-Ravi bulls, corroborating our previous findings [18]. Although cryopreservation consistently reduced total and progressive motility across all genotypes, Nili-Ravi semen had a significant decline in post-thaw total and progressive motility, accompanied by elevated static motility. This suggests a greater vulnerability of Nili-Ravi semen to cryo-damage under our experimental conditions. This reduced post-thaw progressive motility in Nili-Ravi, along with reduced plasma membrane integrity, suggests potential issues like increased fatty acid peroxidation or ROS generation. Sperm motility is intricately influenced by variables including oxidative stress, fatty acid oxidation, metabolism of energy substrates, and concentration of energy substrates in seminal plasma [19,20]. Linear motility, essential for effective AI, is notably enhanced by fatty acid oxidation and ATP synthesis [21]. Although a limitation of our study was the lack of direct measurement for fatty acid peroxidation, mitochondrial membrane potential, and total ROS, these factors might explain the poor post-thaw motility in Nili-Ravi semen.

For a better understanding, we evaluated sperm morphometry and kinematics that provided additional insights into genotypic differences in sperm function. Sperm morphology describes the shape of the sperm, the tail’s structure, the midpiece’s appearance, and the size and DNA content of the head [22]. There is a positive correlation between swimming speed and flagellar length, which allows faster spermatozoa to reach the uterotubal junction before their competitors [23]. Species with high levels of sperm competition are thought to produce longer spermatozoa than other species [23]. Motility plays a significant role in spermatozoa reaching the site of fertilization. Sperm maintain linear motility to reach the site of fertilization. In the oviduct, sperm movement shifts from linear to a zigzag pattern—an essential characteristic of hyperactivation required for successful fertilization of the ovum [24]. This zigzag motility is marked by an increased VCL and a higher ALH with greater ATP demand in the sperm tail [25]. Therefore, the size of the sperm tail might be linked to fertility. Static and non-motile sperm had smaller head widths and head areas than the motile and progressively motile sperm [26].

Our study also showed a smaller head width and head area of static sperm than motile sperm. Spermatozoa with poor motility are detected in the backflow of the female tract within 15 min after insemination [26]. This suggests that highly motile spermatozoa are the preference of the vaginal tract’s early sperm selection process. Spermatozoa in the backflow were smaller (both the head and flagellum) and had different head shapes than spermatozoa in the dosage before insemination [27]. However, there was no correlation between the mean swimming speed of sperm and the mean length of flagellum [28], which reflects the results of the current study, where there were no significant differences among the tail length of static and motile sperm.

Among the genotypes, there were no differences in sperm morphometry in this experiment. There are differences in sperm size and shape among different species [29]. The head length and width of buffalo sperm were 8.33 and 4.32 µm; cattle sperm measured 7.01 and 4.32 µm, and sheep sperm measured 8.94 and 4.59 µm, respectively [29]. Varied observation results were probably due to the differences in measurement methods used. Our study’s findings regarding the morphometry analysis of the buffalo bull sperm were consistent with those of earlier investigations. This is the first report where we characterize the sperm morphometry of the indigenous buffalo bull of Bangladesh. The elevated motility of fresh semen in indigenous bulls was substantially associated with higher values of VAP, VSL, VCL, and LIN in comparison to Murrah and Nili-Ravi bulls. In general, all three groups of semen experienced a reduction in motion characteristics from collection to post-thaw stages. This could be attributed to holding times, equilibration times, and cryo-injury during cryopreservation [30]. The indigenous bull had considerably more straight and linear post-thaw sperm than any other group, but there was no difference in post-thaw VCL among the genotypes. Bull age, sperm maturation level, sperm energy status, viscosity of the medium, osmolarity, and pH could be the cause of variations in motility and/or kinematic parameter values among breeds [31,32].

Polar lipids linked to several proteins are the primary constituent of the lipid bilayers that organize cell membranes [33]. Compared to somatic cells, the lipid content of the plasma membrane in mammalian sperm differs significantly [34]. The most important variables impacting the integrity of the sperm membrane are sperm sorting, sperm selection, and sperm cryopreservation [35]. Nili-Ravi bull semen has lower plasma membrane integrity than Murrah and indigenous bull semen in both fresh and post-thaw stages, and following cryopreservation, all three genotypes have reduced plasma membrane integrity. The plasma membrane integrity of fresh and frozen Nili-Ravi buffaloes was recorded as 69.5% and 49.8% when cryopreserved with androMed extender [36]. Murrah and Indigenous buffalo also lose 27% [18] and 26% [10] membrane integrity when cryopreserved with an androMed extender. The increased loss of membrane integrity in Nili-Ravi bulls may be explained by a reduced cholesterol-to-phospholipid ratio in their plasma membrane, a recognized determinant of resilience to cold shock [35].

Evaluating the frozen semen production capacity of each bull and genotype is crucial for enhancing the efficiency of AI center operations. No significant differences were observed in the total number of frozen semen doses produced per ejaculate across the genotypes; however, Murrah bulls exhibited the maximum production (300.6 ± 94.71 doses/ejaculate), followed by Nili-Ravi and Indigenous bulls. The doses/ejaculates are greater than those reported for other buffalo breeds [37], perhaps due to the enhanced motility and concentration of fresh semen observed in our study. However, keep in mind that the annual production efficiency might be substantially affected by collection intervals, seasonal fluctuations, freezing efficacy, and comprehensive bull management. Despite the evident disparities in several semen quality metrics, no significant variations in bull fertility (pregnancy rates) were observed among the genotypes after the AI of indigenous buffalo cows. The recorded conception rates, falling within the standard 30%–60% range for water buffalo [38], are comparable to or exceed earlier findings [8,39].

Conception largely depends on semen quality, insemination technique, proper timing of AI, and other management and environmental factors [40]. To reduce variations, the same technician inseminated all of the buffalo in our investigation. Study shows that total and progressive motility have a 12.9% contribution to the overall pregnancy in buffalo [8]. While motility might influence overall pregnancy, the ultimate fertility outcome is complex, and other female factors are also involved. Our multiparametric semen quality assessment verifies that genotype affects both fresh and post-thaw sperm motility and kinematics. Interestingly, although statistically non-significant, the marginally elevated fertility rate in the indigenous group (70.37% ± 6.42%) may be associated with their enhanced post-thaw progressive motility and kinematic parameters, indicating that these characteristics could be vital for fertility despite the absence of statistical differences in overall pregnancy rates.

## Conclusion

Buffalo bulls’ genotype can affect a number of semen quality characteristics, including volume, morphology, motility, and post-thaw survivability. Compared to Murrah and Nili-Ravi bulls, native Bangladeshi buffalo bulls had greater plasma membrane integrity, better kinematic qualities, and superior fresh and post-thaw motility. While these differences suggest enhanced sperm characteristics in indigenous bulls, we observed no variations in fertility rates (pregnancy outcomes) among the three genotypes under on-station AI conditions. Future research needs to be carried out to evaluate the efficacy of AI and fertility rate under on-farm conditions.

## List of abbreviations

AI, Artificial insemination; ALH, Amplitude of lateral head displacement; ANOVA, Analysis of Variance; ATP, Adenosine triphosphate; AV, Artificial vagina; BCF, Beat cross-frequency; BLRI, Bangladesh Livestock Research Institute; CASA, Computer-assisted sperm analyzer; Cu, Copper; DNA, Deoxyribonucleic Acid; FAO, Food and Agriculture Organization; Hg, Mercury; HOST, Hypoosmotic Swelling Test; LIN, Linearity; Mn, Manganese; ROS, Reactive Oxygen Species; STR, Straightness; VAP, Average path velocity; VCL, Curvilinear Velocity; VSL, Straight linear velocity; Zn, Zinc.
